# Supplementary material for: Towards single-chip radiofrequency signal processing via acoustoelectric electron–phonon interactions
Source: Nat Commun. 2021 May 13;12:2769. doi: 10.1038/s41467-021-22935-1 (PMC8119416; doi:10.1038/s41467-021-22935-1)
Supplement: Supplementary file 1 — Supplementary Information [file 41467_2021_22935_MOESM1_ESM.docx]

**Supplementary Information**

**Towards single-chip radiofrequency signal processing via acoustoelectric electron-phonon interactions**

**L. Hackett et al.**


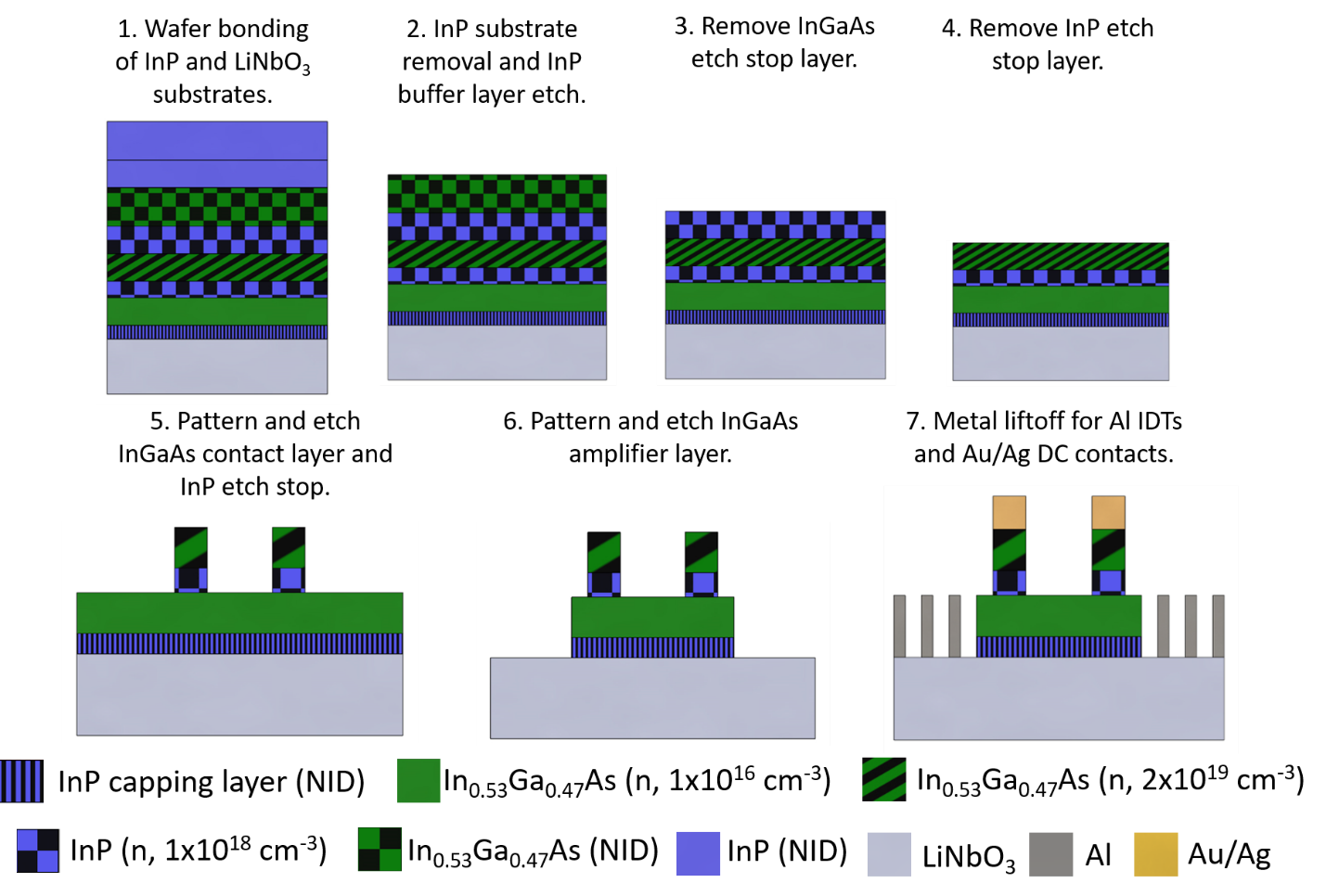


**Supplementary Figure 1.** Fabrication process flow for the acoustoelectric devices.


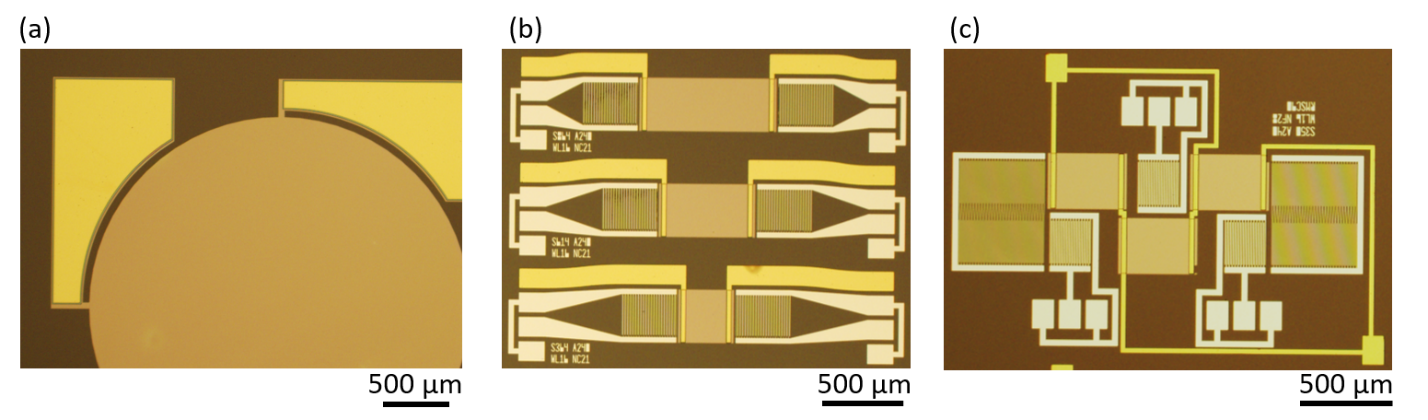


**Supplementary Figure 2.** Optical microscope images of (a) a Hall structure, (b) acoustoelectric amplifiers, and (c) an acoustoelectric circulator after the complete fabrication process.


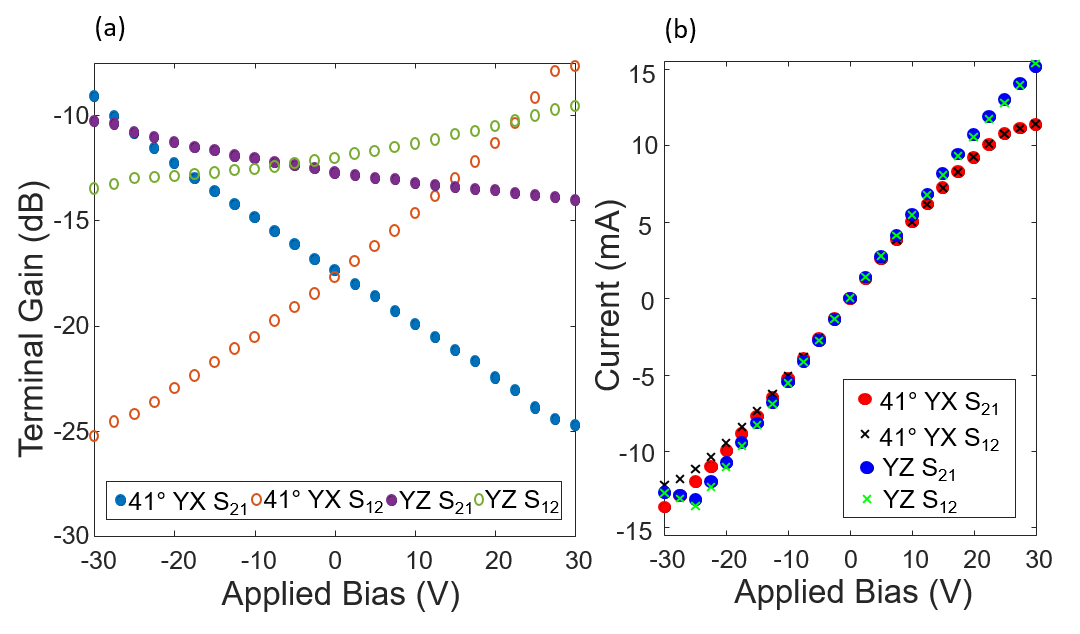


**Supplementary Figure 3.** (a) Terminal gain as a function of applied bias for an acoustoelectric amplifier on 41° YX and YZ LiNbO_3_ substrates. The In_0.53_Ga_0.47_As layer is 300 nm thick and 255 μm long. (b) Current as a function of applied bias taken during the gain measurements.


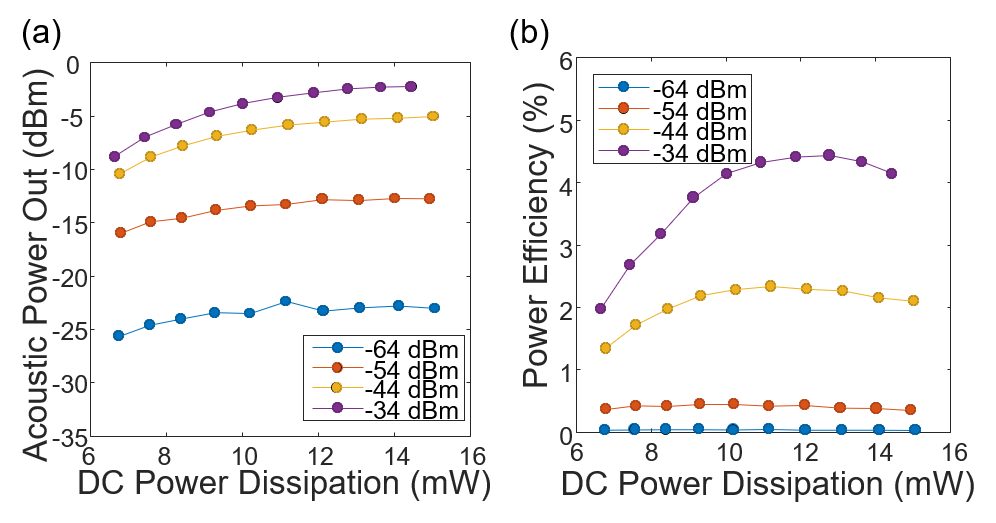


**Supplementary Figure 4.** (a) Acoustic output power as a function of DC power dissipation for various acoustic input powers. (b) DC to acoustic power efficiency as a function of DC power dissipation for various acoustic input powers.


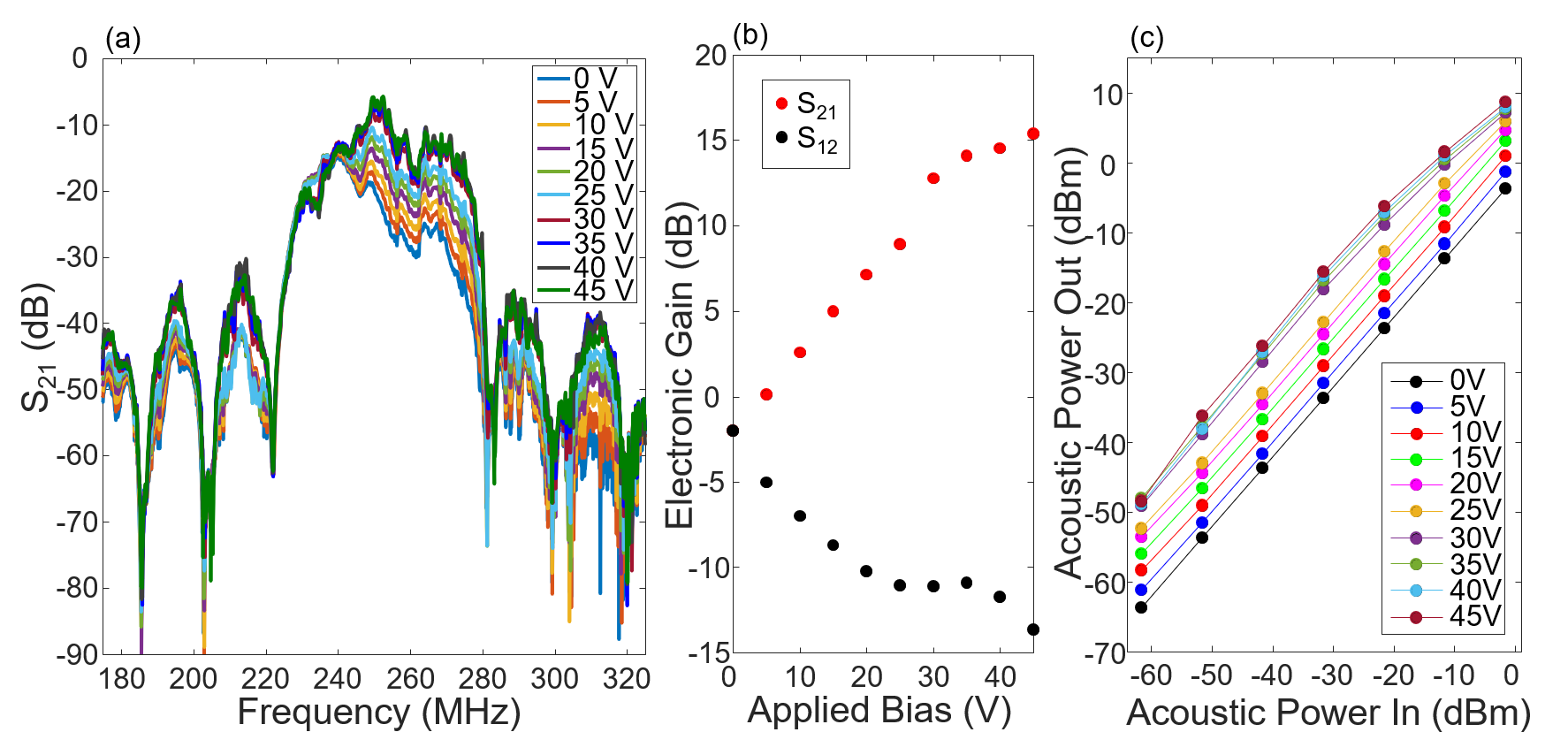


**Supplementary Figure 5.** (a) Measured S_21_ as a function of frequency with increasing applied bias for a 75 nm thick and 250 μm long In_0.53_Ga_0.47_As layer on a 5 μm YX LiNbO_3_ layer on a bulk Si substrate. (b) Electronic gain as a function of applied bias. (c) Acoustic output power as a function of acoustic input power.


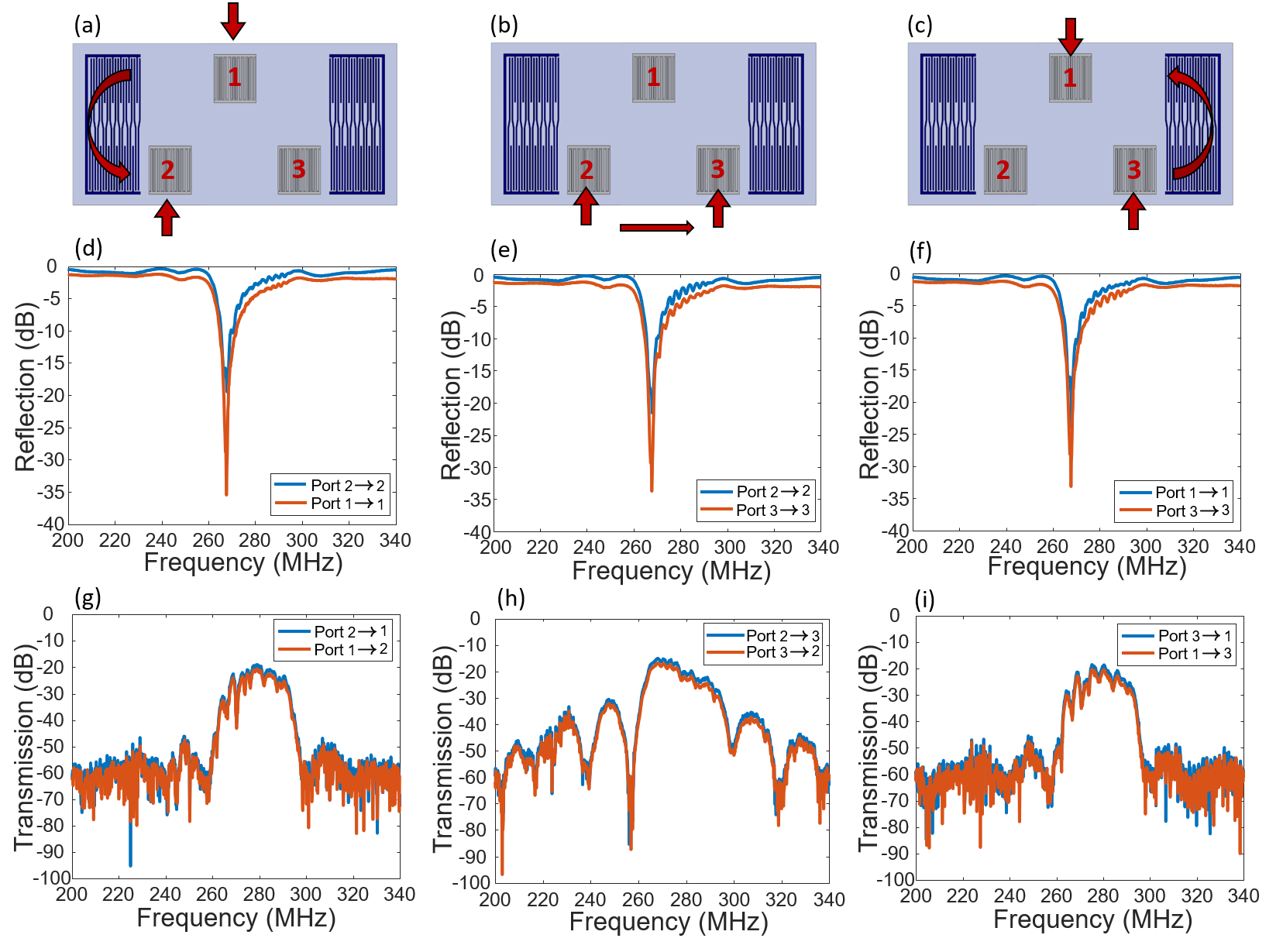


**Supplementary Figure 6.** Configuration to measure S-parameters in a metal only version of the acoustoelectric circulator design from (a) port 1 to port 2 (b) port 2 to port 3, and (c) port 3 to port 1. The corresponding reflection is shown in (d), (e), and (f), respectively while the corresponding transmission is shown in (g), (h), and (i), respectively.

**Supplementary Note 1. Acoustoelectric device fabrication**

The fabrication process flow for the acoustoelectric amplifier is shown in Supplementary Figure 1. The lattice-matched indium gallium arsenide (In_0.53_Ga_0.47_As) and indium phosphide (InP) layer structure is grown by metal-organic chemical vapor deposition (MOCVD) on a 2 inch InP wafer. This epitaxial stack consists of a 500 nm not-intentionally doped (NID) InP buffer layer, a 1500 nm In_0.53_Ga_0.47_As NID etch stop layer, a 100 nm InP etch stop layer doped n-type with silicon (Si) at a concentration of 1x10^18^ cm^-3^, a 100 nm In_0.53_Ga_0.47_As contact layer doped n-type with Si at 2x10^19^ cm^-3^, a 30 nm InP etch stop layer doped n-type with Si at 1x10^18^ cm^-3^, a 50 nm In_0.53_Ga_0.47_As amplifier layer doped n-type with Si at 1x10^16^ cm^-3^, and a 5 nm NID InP capping layer. The InP wafer is then bonded to a 4 inch 41°YX lithium niobate (LiNbO_3_) substrate with the InP capping layer in contact with the LiNbO_3_ surface through a manual initiation followed by annealing at 100°C in vacuum. Wafer bonding is achieved with no noticeable voids due to the smoothness and low defectivity of the epitaxial stack.

Following wafer bonding, the entire InP substrate and buffer layer are etched away in hydrochloric acid (HCl), landing on the In_0.53_Ga_0.47_As NID etch stop layer, which is etched away in a solution of sulfuric acid (H_2_SO_4_), hydrogen peroxide (H_2_O_2_), and water (H_2_O). The 100 nm InP etch stop layer is then etched in a solution of HCl and phosphoric acid (H_3_PO_4_). This is followed by patterning the 100 nm In_0.53_Ga_0.47_As contact layer and etching it in a solution of H_2_SO_4_, H_2_O_2_, and H_2_O followed by etching the 30 nm InP etch stop layer in a mixture of HCl and H_3_PO_4_. The 100 nm In_0.53_Ga_0.47_As contact layer and 30 nm InP etch stop layer provide quasi-Ohmic DC contact between the DC contact metal and the In_0.53_Ga_0.47_As amplifier layer. The 50 nm In_0.53_Ga_0.47_As amplifier layer is then patterned and etched in a H_2_SO_4_, H_2_O_2_, and H_2_O solution. Two metal liftoff steps are then carried out to form the DC contacts and the RF interdigitated transducers (IDTs). The DC contact metal stack is titanium (Ti), gold (Au), silver (Ag), and Au with thicknesses of 20 nm, 100 nm, 1000 nm, and 200 nm, respectively. The metal stack for the IDTs consists of 10 nm chrome (Cr) and 300 nm aluminum (Al). Optical microscope images of a completed Hall structure, amplifier device, and acoustoelectric circulator are shown in Supplementary Figures 2(a-c), respectively. As can be seen, large-area and defect-free In_0.53_Ga_0.47_As on LiNbO_3_ devices are fabricated using this process flow.

**Supplementary Note 2. Properties of commercially available LiNbO_3_ substrates**

Supplementary Table 1 lists commercially available LiNbO_3_ cuts, the acoustic mode of interest, and the propagation loss for the electrically open, or free, boundary condition (α_f_) and the electrically shorted, or metallized, boundary condition (α_m_). The SH-SAW modes supported on LiNbO_3_ have electromechanical coupling coefficient (*K^2^*) values significantly larger than the Rayleigh modes, but they also suffer propagation losses from bulk mode coupling.^1^ Previously we have demonstrated an acoustoelectric amplifier with large electronic gain on YX LiNbO_3_, but large propagation losses for this cut significantly limits the achievable terminal gain and passive delay line performance.^2^ The SH-SAW mode on 41°YX LiNbO_3_ has a large *K^2^* of 17.2% that is significantly larger than the *K^2^* values for Rayleigh modes on YZ and 128° YX LiNbO_3_. In addition, the propagation losses are significantly lower compared to the SH-SAW mode on YX LiNbO_3_.

Supplementary Table 1: LiNbO_3_ wafer properties for available cuts.

| **Cut** | **Mode** | ***K^2^* (%)** | **α_f_ (dB/Λ)** | **α_m_ (dB/Λ)** |
| --- | --- | --- | --- | --- |
| YZ | Rayleigh | 4.9 | 0 | 0 |
| 128° YX | Rayleigh | 5.5 | 0 | 0 |
| YX | SH-SAW | 24.2 | 0.3 | 0.5 |
| 41° YX | SH-SAW | 17.2 | 0 | 0.04 |
| 64° YX | SH-SAW | 11.3 | 0.04 | 0 |

**Supplementary Note 3. Comparison of YZ and 41° YX LiNbO_3_ substrates**

We carried out an initial characterization of the acoustoelectric coupling coefficient ($k_{AE}^{2})$ of the SH-SAW mode on 41°YX LiNbO_3_ with a *K^2^* of 17.2% by comparing the acoustoelectric gain slope to the Rayleigh mode on YZ LiNbO_3_ with a *K^2^* of 4.9%. The In_0.53_Ga_0.47_As on LiNbO_3_ devices were fabricated on the two separate substrates with a 300 nm In_0.53_Ga_0.47_As amplifier layer. The slope of the acoustoelectric gain as a function of drift voltage is expected to depend linearly on $k_{AE}^{2}$. Terminal gain as a function of applied bias is shown in Supplementary Figure 3(a) for a 300 nm In_0.53_Ga_0.47_As amplifier layer on YZ and 41° YX LiNbO_3_. The acoustic wavelength is 16 μm, the IDT aperture is 15Λ, and the acoustoelectric interaction region is 255 μm long. The Rayleigh mode on YZ LiNbO_3_ has a resonant frequency of 215 MHz corresponding to an acoustic velocity of 3440 m/s and the SH-SAW mode on 41° YX LiNbO_3_ has a resonant frequency of 276 MHz, corresponding to an acoustic velocity of 4416 m/s. The acoustoelectric gain slope for the Rayleigh mode on YZ LiNbO_3_ is -0.060122 dB/V and 0.061698 dB/V for the measured S_21_ and S_12_, respectively. For the SH-SAW mode on 41° YX LiNbO_3_, the acoustoelectric gain slope is -0.25746 dB/V and 0.29524 dB/V for the measured S_21_ and S_12_, respectively. Correcting for the frequency dependence of the acoustoelectric gain, the increase in gain slope is at least 3.3X larger for the SH-SAW mode on 41° YX LiNbO_3_ compared to the Rayleigh mode on YZ LiNbO_3_. This agrees well with the expected 3.5X increase in gain slope due to the increase in $k_{AE}^{2}$. The corresponding IV curves for the devices are shown in Supplementary Figure 3(b). The overall conductivity of the In_0.53_Ga_0.47_As amplifier layers on the two different substrates is similar, as expected. There are some nonlinearities in the IV curves with increasing applied bias, which could be due to thermal or stain-induced effects.

**Supplementary Note 4. Acoustic input power dependence and power efficiency**

We measured the acoustic output power and power conversion efficiency as a function of the dissipated DC power for different acoustic input powers. Here we estimate the input acoustic power by taking into consideration the attenuation with no drift field, which is approximately -33 dB. From the experimental gain curve, we find that 5.6 dB is due to loss associated with the acoustoelectric effect according to the calculated voltage to reach the 0 dB operating point based on the device length and the measured semiconductor mobility (*μ*) of 2000 cm^2^/V-s. The remaining 27.4 dB is split between the input and output to account for transducer conversion losses and losses from the DC contacts. By measuring the insertion loss of acoustic delay lines without and with the DC contact metal stack used here, we have found that loss from reflecting from the DC contacts on account of the large impedance change under the thick, high-density metal can be as high as 5 dB per contact. This added insertion loss could be improved by reducing the thickness of the DC contact metal. Here a thickness exceeding 1 μm was used to ensure good Ohmic contact, but since the applied fields are large enough to overcome contact barriers, this is likely not required. The acoustic input power is then the source power with the losses from the input transducer and DC contact while the acoustic power out is the input power with the added electronic gain.

A plot of acoustic output power as a function of the dissipated DC power for different acoustic input powers is shown in Supplementary Figure 4(a) for a 505 μm long amplifier device. Gain rollover can be seen, as there is less change in acoustic output power with increasing dissipated DC power. This is likely due to thermal effects. In addition, gain compression can also be seen as there is less change in the acoustic output power for increasing acoustic input powers. Supplementary Figure 4(b) shows the power efficiency (*η*), defined as $\eta=\frac{P_{OUT}^{A}}{P_{DISS}^{DC}}$, plotted as a function of the dissipated DC power for different acoustic input powers where $P_{OUT}^{A}$ is the acoustic output power and $P_{DISS}^{DC}$ is the dissipated DC power.

**Supplementary Note 5. Noise figure calculation**

The theoretical noise figure $F$, calculated using the impedance field method, is given by

|  | $F=1+\frac{\exp\left( -2\beta h \right)}{{v_{0}}/{v_{a}}-1}\left( \frac{1+{\varepsilon_{p}}/{\varepsilon_{h}\tanh\left( \beta h \right)}}{1+\tanh\left( \beta h \right)} \right)^{2}\left( 1+\frac{D_{TR}}{D_{TH}} \right)\left( \frac{\exp\left( 2\alpha l \right)-1}{\exp\left( 2\alpha l \right)} \right)$ | (S1) |
| --- | --- | --- |

where $l$ is the semiconductor length, $\beta=\omega/{v_{a}}$ is the acoustic wave propagation constant, where $\omega$ is the acoustic wave frequency and $v_{a}$ is the acoustic wave velocity, $h$ is the gap between the semiconductor and the piezoelectric, and $D_{\mathrm{TH}}={kT\mu}/q$ is the thermal diffusion constant where $k$ is Boltzmann’s constant, $T$ is the temperature, $\mu$ is the mobility, and $q$ is the elementary charge. The term $D_{\mathrm{TR}}=\frac{f\left( \omega\right)[1-f\left( \omega\right)]v_{0}^{2}\tau}{1+{(\omega\tau)}^{2}}$ captures the effects of trapping where $f\left( \omega\right)$ is the fraction of the total carriers that are untrapped, $v_{0}$ is the carrier drift velocity, and $\tau$ is the relaxation time for carrier trapping, $\alpha$ is the incremental acoustic gain, $\varepsilon_{p}$ is the piezoelectric permittivity, and $\varepsilon_{h}$ is the gap permittivity.^3^

For the case where the semiconductor is in direct contact with the piezoelectric, Equation S1 reduces to

|  | $F=1+\frac{1}{{v_{0}}/{v_{a}}-1}\left( 1+\frac{D_{TR}}{D_{TH}} \right)\left( \frac{\exp\left( 2\alpha l \right)-1}{\exp\left( 2\alpha l \right)} \right).$ | (S2) |
| --- | --- | --- |

In addition, for the case where the trap density is very small, $f\left( \omega\right)=1$ and Equation S2 futher reduces to

|  | $F=1+\frac{1}{{v_{0}}/{v_{a}}-1}\left( \frac{\exp\left( 2\alpha l \right)-1}{\exp\left( 2\alpha l \right)} \right).$ | (S3) |
| --- | --- | --- |

For our experimental results, we have ${v_{0}}/{v_{a}}=5.4$, $l=550 \mu m$, and $\alpha=806 dB/cm$. Plugging in these values to Equation S3, we obtain $F=1.23$.

**Supplementary Note 6. Acoustic wave amplifier operating continuously on a LiNbO_3_ on Si substrate**

The potential to improve the thermal management of active acoustic wave devices in our material platform was assessed by replacing the 41°YX LiNbO_3_ substrate with a substrate consisting of 5 μm thick film of YX LiNbO_3_ on bulk Si. The difference in acoustic velocity between Si and LiNbO_3_ causes a launched acoustic wave to be guided in the LiNbO_3_ film. Si has a thermal conductivity of 150 W/m-K while LiNbO_3_ has a thermal conductivity of 4.6 W/m-K.^4,5^ Therefore, it is expected that heat dissipation will be significantly improved through the 30X increase in thermal conductivity for Si over LiNbO_3_. Supplementary Figure 5(a) shows the measured S_21_ as a function of frequency with increasing applied bias for a 250 μm long device with a 75 nm thick In_0.53_Ga_0.47_As amplifier layer with a measured Hall mobility of 2360 cm^2^/V-s and a doping concentration of 1.9x10^16^ cm^-3^. The device shows stable operation with a continuously applied DC bias. Supplementary Figure 5(b) shows the electronic gain as a function of applied bias. An electronic gain of 15.4 dB is achieved with an applied bias of 45 V. Electronic gain occurs in these devices when the amplification is large enough to overcome the losses due to the acoustoelectric effect. Gain only occurs when the electron drift velocity exceeds the acoustic velocity and therefore a voltage must be applied to reach 0 dB of electronic gain. The value of this voltage depends on the device length and the semiconductor mobility. For this device the required applied bias to reach 0 dB of electronic gain is 4V. Therefore, the electronic gain is defined based on the subtraction of the insertion loss at 253 MHz with an applied bias of 4V and 45V. As can be seen, there is a corresponding attenuation of 13.6 dB for the backward propagating wave. Although this device did not have as high of a gain performance as the devices presented in the main text, this is attributed to differences in the epitaxial layer that we can amend in the future and thus have both thermal dissipation advantages and exceptional gain performance in the same device. Supplementary Figure 5(c) shows the acoustic input power as a function of the acoustic output power. The device shows stable performance for the range of tested acoustic input powers, up to -1.6 dBm, and a linear response up to an acoustic output power of 6 dBm.

**Supplementary Note 7.** **S-parameters of reversing multistrip couplers**

In order to better assess the acoustoelectric circulator performance and the correct path forward to improve insertion loss, we evaluated a metal-only three port ring filter on 41° YX LiNbO_3_ and characterized the pairs of ports as shown in Supplementary Figure 7(a-c). The reflection data as a function of frequency for ports 1 and 2, ports 2 and 3 and ports 3 and 1 are shown in Supplementary Figures 7(d-f), respectively while the corresponding transmission data as a function of frequency are shown in Supplementary Figures 7(g-i), respectively. From port 1 to port 2 and from port 3 to port 1 the acoustic wave must pass through the reversing multistrip coupler (RMSC) and the corresponding insertion loss is 19 dB. From port 2 to port 3, where there is no RMSC, the insertion loss is 15 dB. These results suggest that improvement to the acoustoelectric circulator performance, in terms of insertion loss, can be achieved through a more sophisticated electromechanical transducer design and optimized RMSCs.

**References**

1 Adler, E. L. SAW and Pseudo-SAW Properties Using Matrix-Methods. *IEEE T Ultrason Ferr* **41**, 876-882, (1994).

2 Hackett, L. *et al.* High-gain leaky surface acoustic wave amplifier in epitaxial InGaAs on lithium niobate heterostructure. *Appl Phys Lett* **114**, (2019).

3 Kino, G. S. & Coldren, L. A. Noise Figure Calculation for Rayleigh-Wave Amplifier. *Appl Phys Lett* **22**, 50-52, (1973).

4 Glassbrenner, C. J. & Slack, G. A. Thermal Conductivity of Silicon + Germanium from 3 Degrees K to Melting Point. *Phys Rev* **134**, 1058, (1964).

5 Wong, K.-K. *Properties of lithium niobate*. (IET, 2002).
